# Identifying novel clinical phenotypes of acute respiratory distress syndrome using trajectories of daily fluid balance: a secondary analysis of randomized controlled trials

**DOI:** 10.1186/s40001-024-01866-9

**Published:** 2024-05-28

**Authors:** Fei Wu, Suqin Shi, Zixuan Wang, Yurong Wang, Le Xia, Qingling Feng, Xin Hang, Min Zhu, Jinqiang Zhuang

**Affiliations:** 1 Department of Emergency Intensive Care Unit (EICU), The Affiliated Hospital of Yangzhou University, Yangzhou University, No. 45 Taizhou Road, Guangling District, Yangzhou City, 225000 Jiangsu Province China; 2 School of Nursing, School of Public Health, Yangzhou University, No. 136 Jiangyang Middle Road, Yangzhou, 225009 Jiangsu China

**Keywords:** Acute respiratory distress syndrome, Phenotype, Joint latent class mixed model, Fluid balance

## Abstract

**Background:**

Previously identified phenotypes of acute respiratory distress syndrome (ARDS) could not reveal the dynamic change of phenotypes over time. We aimed to identify novel clinical phenotypes in ARDS using trajectories of fluid balance, to test whether phenotypes respond differently to different treatment, and to develop a simplified model for phenotype identification.

**Methods:**

FACTT (conservative vs liberal fluid management) trial was classified as a development cohort, joint latent class mixed models (JLCMMs) were employed to identify trajectories of fluid balance. Heterogeneity of treatment effect (HTE) for fluid management strategy across phenotypes was investigated. We also constructed a parsimonious probabilistic model using baseline data to predict the fluid trajectories in the development cohort. The trajectory groups and the probabilistic model were externally validated in EDEN (initial trophic vs full enteral feeding) trial.

**Results:**

Using JLCMM, we identified two trajectory groups in the development cohort: Class 1 (*n* = 758, 76.4% of the cohort) had an early positive fluid balance, but achieved negative fluid balance rapidly, and Class 2 (*n* = 234, 24.6% of the cohort) was characterized by persistent positive fluid balance. Compared to Class 1 patients, patients in Class 2 had significantly higher 60-day mortality (53.5% vs. 17.8%, *p* < 0.001), and fewer ventilator-free days (0 vs. 20, *p* < 0.001). A significant HTE between phenotypes and fluid management strategies was observed in the FACTT. An 8-variables model was derived for phenotype assignment.

**Conclusions:**

We identified and validated two novel clinical trajectories for ARDS patients, with both prognostic and predictive enrichment. The trajectories of ARDS can be identified with simple classifier models.

**Supplementary Information:**

The online version contains supplementary material available at 10.1186/s40001-024-01866-9.

## Introduction

Acute respiratory distress syndrome (ARDS) is a heterogenous syndrome characterized by acute hypoxic respiratory failure that can be caused by a wide variety of insults [1]. The diverse populations, multiple etiologies, and a broad definition lend to the clinical heterogeneity of ARDS, which might explain the absence of benefit in most randomized controlled trials (RCTs) assessing various treatment strategies [2]. Identification of specific ARDS phenotypes could lead to more positive clinical trials and personalized ARDS management.

Several ARDS phenotypes have been documented [3]. Using clinical data from negative RCTs of ARDS, two phenotypes have been identified. Compared to patients with the hypo-inflammatory phenotype, those with the hyper-inflammatory phenotype had higher levels of pro-inflammatory biomarkers and poorer outcomes, subsequent analyses demonstrated that patients with the hyper-inflammatory phenotype could benefit more from higher positive end-expiratory pressure (PEEP) and conservative fluid strategy [4, 5]. However, these phenotypes do not capture the complexity and diversity of ARDS, and, because they are based on data collected within the first day do not reveal dynamic changes in the patients’ conditions over time.

Daily fluid balance is a dynamic variable that changes throughout the course of a patient’s illness, and varies across patients. Cumulative positive fluid balance was associated with increased duration of mechanical ventilation (MV) and mortality in ARDS patients, presumably as a marker of worsening pulmonary edema, deteriorating organ function and respiratory mechanics [6–8], and conservative fluid management was associated with improved organ function and decreased intensive care unit (ICU) length of stay [9]. However, persistent negative fluid balance could induce acute circulatory failure in patients with sepsis related ARDS. We assumed that the trajectories of fluid balance indicate the dynamic change of illness over time, which might be a helpful to classify dynamic phenotypes of ARDS patients in clinical.

Therefore, we designed a secondary analysis to identify trajectories of fluid balance in ARDS patients. We hypothesized that each of trajectory groups would have different demographics, physiological characteristics, mortality rate, and most importantly, respond differently to treatments. We also aimed to derive and validate a simplified probabilistic model for phenotype assignment.

## Methods

### Study design and participants

Patients for this secondary analysis were drawn from two randomized clinical trials from the National Heart, Lung, and Blood Institute (NHLBI) ARDS Network: FACTT (conservative vs liberal fluid management) [10], and EDEN (initial trophic vs full enteral feeding) [11]. The study designs and patient characteristics were available in the original reports. Briefly, FACTT cohort comprised 1000 adult patients with ARDS between 2000 and 2005, and was classified as a development cohort in the present study. We used the EDEN cohort which enrolled 1000 adult patients with ARDS admitted to 44 hospitals between 2008 and 2011 as an external validation cohort. Patients in both RCTs were all intubated and received invasive MV. We excluded patients who had no data of fluid balance within 7 days after randomization.

All data were obtained and approved by Biologic Specimen and Data Repository Information Coordinating Center (BioLINCC, https://biolincc.nhlbi.nih.gov). STROBE recommendations were followed.

### Data collection and outcomes

The detail of data collection is presented in the supplement. The primary outcome in the FACTT cohort was 60 days mortality, and was ventilator-free days (VFDs) to 28 days in the EDEN cohort (details in Additional file 1).

### Phenotype derivation

In the development cohort, joint latent class mixed models (JLCMM) was used to identify novel phenotypes based on daily fluid balance (D0–D7). JLCMM considers the population of subjects as heterogeneous, and assumes that it consists of homogeneous latent subgroups of subjects that share the same marker trajectory (fluid balance) and the same risk of the event (mortality) (details in Additional file 1) [12]. Based on the previous studies identified two to four phenotypes in sepsis and ARDS cohorts, we estimated models ranging from two to five classes [13, 14]. The best-fit GBTM model was selected based on the Bayesian information criterion (BIC), and the minimum number of patients should be over 5% of the entire study population [15]. The trajectories of fluid balance were also validated in the EDEN cohort.

### Statistical analyses

Values are presented as proportions for categorical variables and means (standard deviations) or medians [interquartile ranges (IQRs)] for continuous variables. For comparisons, we used analysis of variance and the Kruskal–Wallis test for continuous data and the *X*^2^ test for categorical data.

We first compared 28-day mortality of patients in the different phenotypes in the development cohort with Kaplan–Meier curves and log-rank tests; we also performed a multivariate Cox regression model to explore the relationship between phenotypes and the 28-day mortality, and adjusted for age, sex and body mass index (BMI).To avoid bias induced by missing data (Additional file 1: Tables S1, S5), we imputed the missing data prior to Cox regression.

Heterogeneity of treatment effect (HTE) was evaluated by the interaction test to determine if treatment effects were differential across phenotypes in FACTT and EDEN cohort, respectively. HTE was assessed by the interaction term (class × treatment strategy) of the Cox regression for mortality and Poisson regression for VFDs.

We also attempted to construct a parsimonious model to predict phenotypes using baseline variable (D0). Gradient boosted model (GBM) was used to identify the most critical classifier variables, and we further selected the top eight variables to construct a phenotype prediction model using GBM. The ability of the final model to predict the phenotypes as identified by calculating the area under the receiver operating characteristic curves (AUROC).

Lastly, to investigate whether we can detect a differential effect of treatment when phenotype is defined by the prediction model rather than the JLCMM, we repeat the HTE analysis using the phenotypes as defined by the prediction model.

The *p*-value was calculated to evaluate the differences between phenotypes, and *p* < 0.05 was considered statistically significant. All statistical analyses were performed using R (version 4.0.3).

## Results

### Derivation of fluid balance trajectories of ARDS in FACTT cohort

**Fig. 1:**
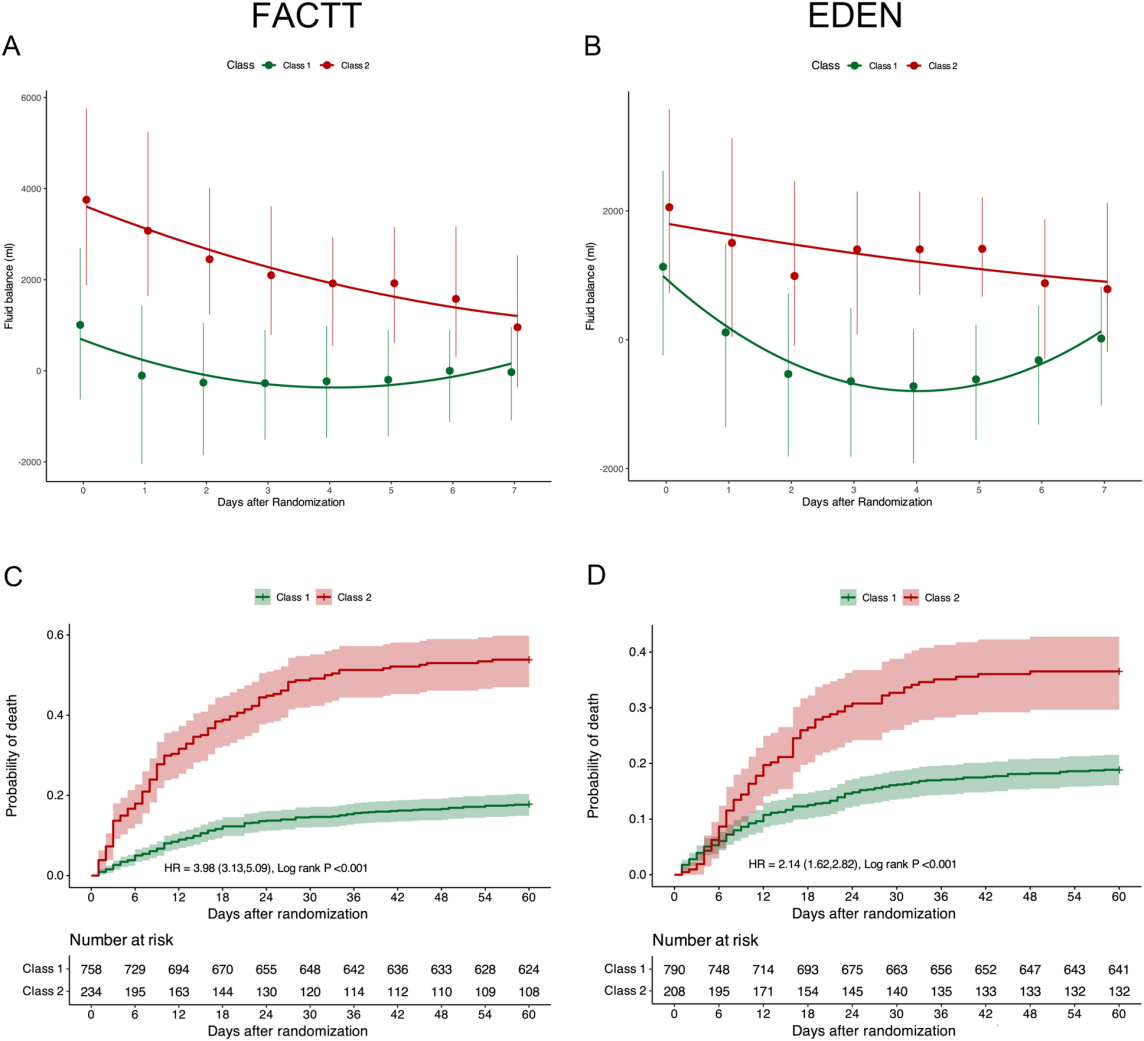
Derivation of phenotypes of ARDS based on joint latent class mixed model (JLCMM) and clinical outcomes between phenotypes. Using JLCMM, two phenotypes were identified in the development (**A**) and validation (**B**) cohorts, **C** shows the Kaplan–Meier survival curves to day 60 according to phenotypes in the development cohort. **D** Shows the Kaplan–Meier survival curves to day 60 according to phenotypes in the validation cohort

The development cohort consisted of 992 ARDS patients from FACTT cohort. Using JLCMM, a model with two distinct trajectory groups were identified and had the optimal fit (Fig. 1A, Additional file 1: Table S2). Class 1 (*n* = 758, 76.4% of the cohort) had an early positive fluid balance, but achieved negative fluid balance rapidly, and Class 2 (*n* = 234, 24.6% of the cohort) was characterized by persistent positive fluid balance.

**Fig. 2:**
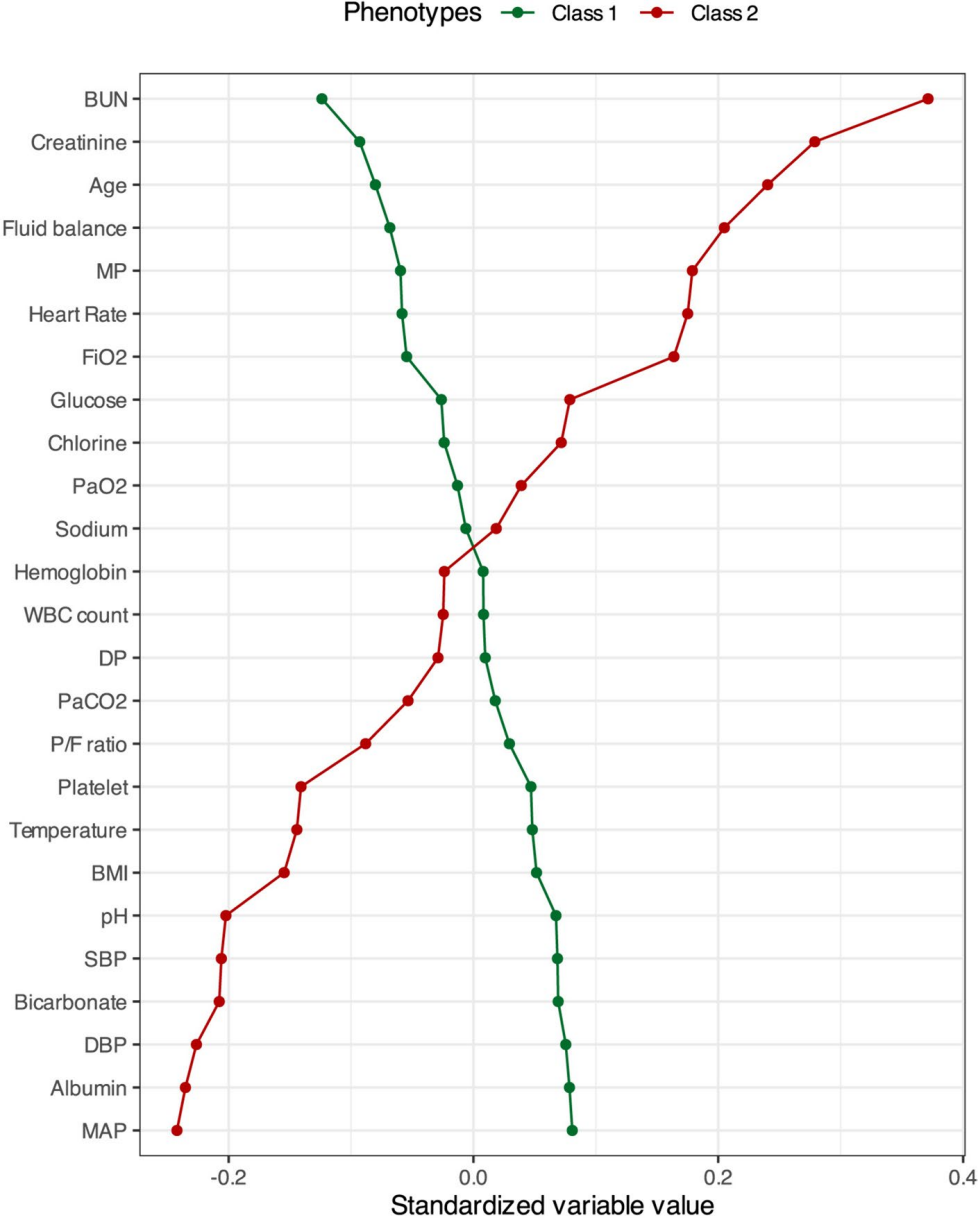
Standardized mean differences between three two trajectory phenotypes in the development cohort. The continuous variables are standardized such that all means are scaled to 0 and SDs to 1. *BUN* blood urea nitrogen, *MP* mechanical power, *FiO*_*2*_ fraction of inspired oxygen, *PaO*_*2*_ partial pressure of oxygen, *DP* driving pressure, *PaCO*_*2*_ partial pressure of carbon dioxide, *P/F ratio* partial pressure of oxygen/ fraction of inspired oxygen ratio, *BMI* body mass index, *SBP* systolic blood pressure, *DBP* diastolic blood pressure, *MAP* mean arterial pressure

Baseline patient characteristics for the two phenotypes are presented in Table 1 and Fig. 2. Compared to patients in Class 1, patients in Class 2 had higher respiratory rate, blood urea nitrogen, and creatinine, but lower mean arterial blood pressure, bicarbonate and platelet. There were statistically significant differences in vasopressor use and in ARDS risk factors between the two phenotypes. Notably, patients classified within Class 2 exhibited a higher incidence of vasopressor requirement and were more commonly diagnosed with sepsis upon study enrollment.

**Table 1:** Comparison of baseline characteristics and outcomes between two phenotypes in FACTT cohort

Characteristics	All (*n* = 992)	Trajectories of fluid balance		
		Class 1 (*n* = 758)	Class 2 (*n* = 234)	*p* value
Age (years)	49 (38, 60)	47 (37, 59)	54 (41, 65)	< 0.001
Male (gender), *n* (%)	532 (53.6)	393 (51.8)	139 (59.4)	0.0051
BMI (kg/m^2^)	27.3 (23.2, 32.5)	27.7 (23.9, 32.9)	25.7 (21.6, 31.1)	< 0.001
ARDS primary risk factor, *n* (%)				< 0.001
Pneumonia	469 (47.3)	349 (46)	120 (51.3)	
Sepsis	230 (23.2)	160 (21.1)	70 (29.9)	
Aspiration	148 (14.9)	127 (16.8)	21 (9)	
Other	145 (14.6)	122 (16.1)	23 (9.8)	
Charlson comorbidity index	0 (0, 2)	0 (0, 2)	1 (0, 3)	0.031
APACHE III	91 (71, 116)	87 (66, 109)	105 (86, 129)	< 0.001
Severity of ARDS at baseline, *n* (%)				0.003
Mild	207 (21.7)	157 (21.5)	50 (22.5)	
Moderate	496 (52)	400 (54.7)	96 (43.2)	
Severe	250 (26.2)	174 (23.8)	76 (34.2)	
Parameters of mechanical ventilation in the first 24 h				
Respiratory rate (breaths min^−1^)	25 (20, 31)	24 (19, 30)	27 (21, 32)	< 0.001
Tidal volume (mL/kg PBW)	6.2 (6.0–7.4)	6.3 (6.0–7.6)	6.1 (6.0–7.3)	0.816
Minute ventilation (L/min)	11.9 (9.5, 14.7)	11.6 (9.34, 14.3)	13 (9.9, 15.6)	< 0.001
PEEP (cmH_2_0)	10 (8, 12)	10 (8, 12)	10 (8, 12)	0.446
Plate Pressure (cmH_2_0)	25 (21, 29)	25 (21, 29)	26 (21, 29)	0.393
Driving pressure (cmH_2_0)	15 (12, 18)	15 (12, 18)	15 (12, 19)	0.71
Mechanical power (J/min)	23.2 (16.7, 32.3)	22.6 (16.6, 31.6)	25.9 (17.3, 34.3)	0.012
PaCO_2_ (mmHg)	39 (34, 45)	39 (34, 45)	39 (32, 45)	0.089
PaO_2_/FiO_2_ ratio (mmHg)	140 (99, 193.)	144 (103, 193)	128 (90, 193)	0.014
Vasopressor use in the first 24 h, *n* (%)	325 (32.8)	223 (29.4)	102 (43.6)	< 0.001
Vital signs in the first 24 h				
Heart rate (beats min^−1^)	102 (87, 117)	100 (86, 115)	107 (91, 120)	0.002
MAP (mmHg)	75 (67, 86)	76 (68, 87)	71 (65, 82)	< 0.001
CVP (mmHg)	12 (9, 15)	11 (8, 14)	12 (9, 15)	0.044
Temperature (℃)	37.5 (36.9, 38.2)	37.6 (36.9, 38.2)	37.4 (36.6, 38.3)	0.129
Laboratory data in the first 24 h				
pH	7.37 (7.3, 7.43)	7.37 (7.31, 7.43)	7.36 (7.27, 7.41)	0.007
BUN (mg/dL)	18 (12, 29.25)	16 (11, 26)	24 (16, 43)	< 0.001
Creatinine (mg/dL)	1 (0.7, 1.5)	0.9 (0.7, 1.4)	1.3 (0.8, 1.9)	< 0.001
Bicarbonate (mmol/L)	22.2 (19, 26)	23 (19, 26)	21 (17, 25)	< 0.001
Platelet (× 10^9^/L)	183 (106, 261)	188 (110, 263)	164 (92, 249)	0.028
Fluid balance in the first 24 h (L)	1.91 (0.43, 4.29)	1.85 (0.34, 3.88)	2.28 (0.83, 5.10)	0.001
Alive and VFDs at day 28 (days)	18 (0, 23)	20 (10, 23)	0 (0, 16)	< 0.001
60-day mortality, *n* (%)	261 (26.3)	135 (17.8)	126 (53.8)	< 0.001

*BMI* body mass index, *ARDS* acute respiratory distress syndrome, *APACHE III* Acute Physiology and Chronic Health Evaluation III, *PBW* predicted body weight, *PEEP* positive end-expiratory pressure, *PaCO*_*2*_ partial pressure of carbon dioxide, *PaO*_*2*_ partial pressure of oxygen, *FiO*_*2*_ fraction of inspired oxygen, *MAP* mean arterial blood pressure, *CVP* central venous pressure, *BUN* blood urea nitrogen, *VFD* ventilator-free day

### Comparisons of clinical outcomes between phenotypes

In the FACTT cohort, patients assigned to Class 2 had significantly higher 60-day mortality (53.5% vs. 17.8%, *p* < 0.001), and fewer ventilator-free days (0 vs. 20, *p* < 0.001) compared to patients assigned to Class 1 (Table 1). Kaplan–Meier survival curves also showed the 60-day mortality was highest in Class 2 patients compared to Class 1 patients (*p* < 0.001) (Fig. 1C).

### Heterogeneity of treatment effect within phenotypes in the FACTT cohort

**Fig. 3:**
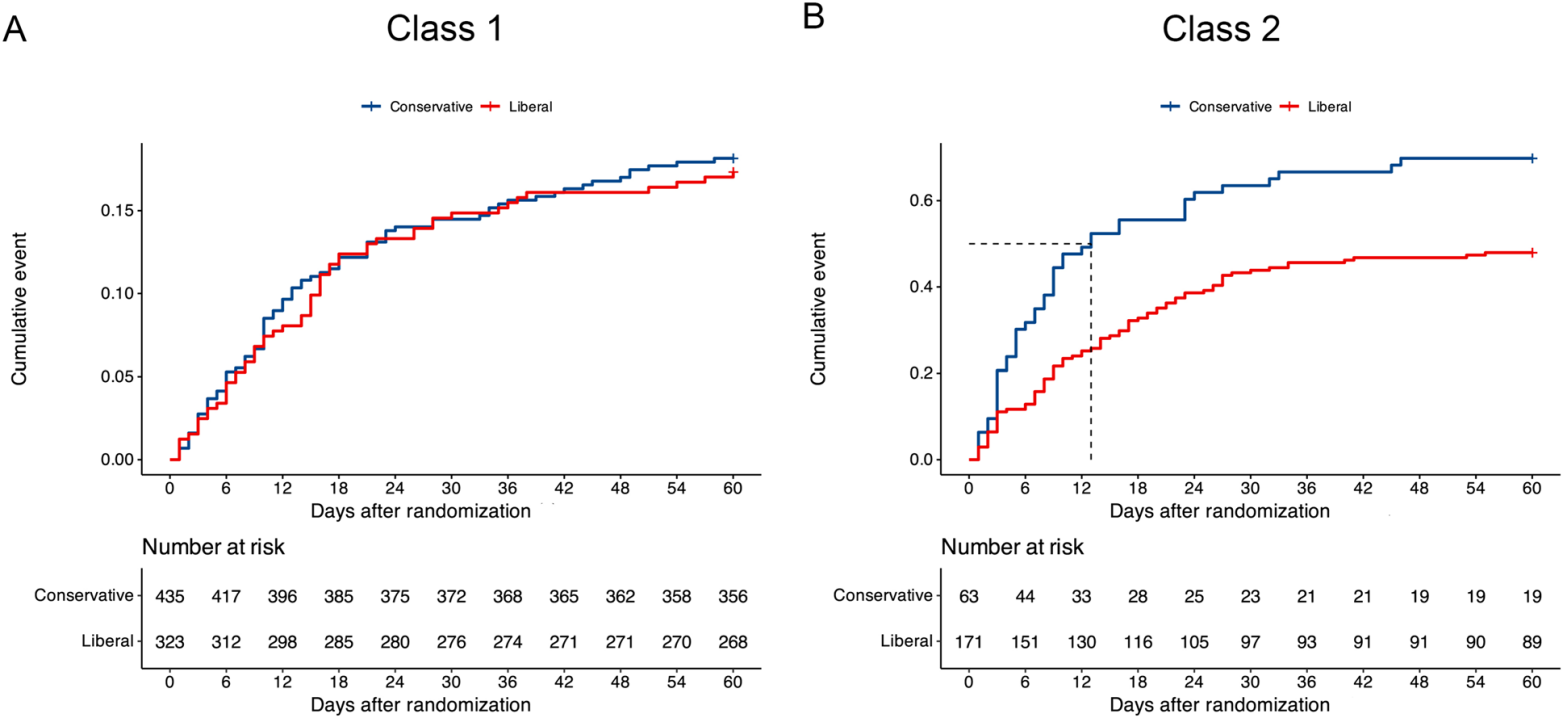
Interaction between phenotypes and fluid management strategy on 60-day mortality in FACTT cohort. **A** The effect of fluid management strategy on 60-day mortality in patients assigned to Class 1; **B** The effect of fluid management strategy on 60-day mortality in patients assigned to Class 2

We detected a significant interaction between phenotypes and fluid management strategy on 60-day mortality. Specifically, mortality among Class 1 patients was 18.2% with fluid conservative strategy compared to 17.0% with the fluid liberal strategy. While mortality among Class 2 patients was 69.8% with the fluid conservative strategy compared to 47.9% with the fluid liberal strategy (*p* for interaction = 0.033) (Table 2; Fig. 3). No significant HTE was observed for ventilator-free days.

**Table 2:** Differences in response to fluid management strategy by phenotypes (FACTT cohort)

Fluid-management strategy	Class 1 (*n* = 758)		Class 2 (*n* = 234)		*p* value for interaction
	Conservative (*n* = 435)	Liberal (*n* = 323)	Conservative (*n* = 63)	Liberal (*n* = 171)	
60-day mortality, *n* (%)	18.2	17.0	69.8	47.9	0.033
Ventilator-free days, days	21 (12, 24)	19 (10, 23)	0 (0, 10)	0 (0,16)	0.106

### Parsimonious probabilistic model can identify trajectory of fluid balance

The most important classifier variables for predicting phenotypes are shown in Additional file 1: Fig. S1. The top eight variables were blood urea nitrogen, BMI, albumin, fluid balance within 24 h prior to randomization, central venous pressure, *P*/*F* ratio, peak pressure and chlorine, which were further utilized to construct the prediction model. The probabilistic model had an AUROC of 0.80 (95% CI 0.77–0.83)(Additional file 1: Fig.S2). Using Youden’s index, we found that the model identified Class 1 with greater accuracy as compared to Class 2 in the FACTT cohort (Additional file 1: Table S3).

We further explore the utility of the probabilistic model, we re-analyze the HTE between fluid management strategy and phenotypes as identified by this model, although mortality among Class 2 patients was 69.1% with the fluid conservative strategy compared to 48.3% with the fluid liberal strategy, the interaction was not statistically significant (*p* for interaction = 0.07) (Additional file 1: Table S4).

### Validation of phenotypes and the probabilistic model in EDEN cohort

The validation cohort consisted of 998 ARDS patients from EDEN cohort. Similar to the development set, two distinct trajectory groups were identified using JLCM (Fig. 1B; Additional file 1: Table S6): Class 1 (*n* = 790, 79.2% of the cohort) and Class 2 (*n* = 208, 20.8% of the cohort). The baseline characteristics for patients in the two phenotypes of the validation cohort are summarized in Additional file 1: Table S7. Patients assigned to Class 2 had significantly fewer ventilator-free days (0 vs. 21, *p* < 0.001), and higher 60-day mortality (36.5% vs. 18.9%, *p* < 0.001) (Fig. 1D). No significant HTE was observed in the EDEN cohort (Additional file 1: Table S8). Similar to the development set, the probabilistic model identified Class 1 with greater accuracy as compared to Class 2 in the EDEN cohort (Additional file 1: Table S9).

## Discussion

The novel findings of our analyses can be summarized as follows. First, based on the trajectories of daily fluid balance, we identified two novel clinical trajectories of ARDS with different demographics, physiological characteristics, and mortality. Second, patients with different fluid trajectories of ARDS responded differently to fluid management strategy. Third, we developed a simplified probabilistic model to predict phenotypes of ARDS, and which is also potentially applicable to another cohort. These findings indicate that identify and target distinct dynamic phenotypes of ARDS might be fundamentally important in future ARDS clinical trials.

Clinical phenotypes have been described for patients with ARDS, and could lead to more personalized management strategies. However, these phenotypes based only on cross-sectional data collected within 1 day, while the phenotypes might be dynamic and change throughout the course of a patient’s illness. A retrospective study divided septic patients into four illness categories based on severity of laboratory and vital sign abnormalities, and declared that almost 60% of the septic patients changed their illness category at least once during hospitalization [16]. To our best knowledge, whether the phenotypes of ARDS are dynamic and follow distinct trajectories over time have never been explored.

In our study, using JLCMM, we discovered and validated two groups of ARDS patients that follow distinct trajectories of fluid balance, and with both prognostic and predictive enrichment. Similar approach has been used in limited studies. According to 986 patients with septic shock, Wang et al. [17] identified three latent fluid balance trajectories using GBTM, which included decreased, low and high fluid balance, and the decreased Fluid Balance trajectory was associated with a low risk of hospital mortality. A prospective study also showed the trajectories of fluid balance in patients undergoing cardiac and aortic surgery, which were significantly associated with risk of acute kidney injury and dialysis [18].

We choose fluid balance to classify ARDS patients for a few reasons. First, fluid balance was associated with clinical outcomes in ARDS. An observational cohort study showed that ARDS patients with a higher cumulative fluid balance on day 7 had a longer length of ICU stay and shorter VFDs, further analysis found that a more positive fluid balance predicted mortality and a negative fluid balance indicated a trend towards survival [6]. Another study declared that time-varying fluid balance predict the 6-month death in ARDS patients [19]. Second, fluid balance or fluid overload might influence the respiratory mechanics. Compared to the liberal fluid strategy in ARDS, patients in the conservative group had a better oxygenation, lung compliance, and a lower plateau pressure during MV within the first 7 days after randomization [10]. We also found that patients in Class 2 (persistent positive fluid balance) were accomplished with higher mechanical power, respiratory rate and lower P/F ratio. Third, positive fluid balance was correlated with excessive inflammation in ARDS. Based on pediatric patients with ARDS, a prospective observational study demonstrated that day 1 plasma interleukin-6 levels were associated with the development of day 3 positive cumulative fluid balance [20]. Although the inflammatory biomarkers were not measured in present study, but because patients in Class 2 presented with persistent positive fluid balance and high proportion of vasopressor use, we hypothesized that Class 2 patients were concomitant with an excessive persistent inflammatory status. Future studies need to investigate the direct correlations between fluid balance trajectories and inflammation.

Classification of patients into phenotypes is aimed to design personalized treatment strategies. The original FACTT trial found no difference in 60-day mortality between conservative and liberal fluid management [21]. After that, Famous et al. discovered that the conservative strategy was associated with improved mortality in patients with hyperinflammatory phenotype but had the opposite effect in patients with hypoinflammatory phenotype [5]. Similar to the previous study, we declared that fluid management strategies had no effect in Class 1 patients, while Class 2 patients can particularly benefit from the liberal fluid strategy. As we discussed above, Class 2 may represent a persistent inflammatory response and organ failure, which was similar to the hypoinflammatory phenotype.

Our study is the first to identify four novel dynamic clinical phenotypes of ARDS using trajectories of fluid balance in NHLBI ARDS Network datasets, and validated in a separate cohort. In addition, both the two cohorts represent a large sample size for ARDS clinical trials. This study also has important limitations. First, although we identified and validated the fluid balance trajectories in two separate cohorts, several factors might affect the fluid balance trajectories [22], such as the missing data, the use of diuretic and renal replacement therapy. Meanwhile, whether these two phenotypes persist in the real-world clinical setting remains unclear. Future researches need to explore the generalization of fluid balance trajectories in addition ARDS cohorts. Second, although numerous studies declared the deteriorating effect of positive fluid balance, the fluid balance targets are typically set during clinical rounds and monitored closely, which may reflect the quality of clinical decision-making and execution rather than the illness state itself. Third, our study represents a secondary analysis of randomized controlled trials, where potential imbalances in baseline characteristics may exist between conservative and liberal fluid management strategies across the identified trajectory groups. Additionally, the use of trajectory-based phenotypes in treatment effect analysis could potentially introduce bias. Further investigation is essential to comprehensively evaluate the effects of this methodology on the heterogeneity of treatment effects. Finally, we developed a simplified probabilistic model to predict the fluid balance trajectories, but the predictive value is relatively low in certain trajectory, which might limit its clinical utility. The main interpretation is that the fluid balance trajectories are dynamic, the predictive value of static values is limited. Future study needs to construct a more simplified and dynamic model to differentiate fluid balance trajectories.

## Conclusion

In conclusion, the secondary analysis of FACTT and EDEN has discovered two novel fluid balance trajectory phenotypes of ARDS patients. In FACTT, patients with persistent positive fluid balance could benefit more from liberal fluid strategy. We also developed a simplified probabilistic model for patient assignment to phenotypes.

### Supplementary Information

**Additional file 1: Table S1.** Percentage of missing data in the variables of interest in FACTT cohort. **Table S2.** BIC Fit statistics and model selection in the FACTT cohort. **Table S3.** Accuracy of a Parsimonious probabilistic model in Correctly Identifying ARDS trajectories in FACTT cohort. **Table S4.** Interaction between ARDS phenotypes as defined by Probabilistic model and fluid management strategy for 60-day mortality in FACTT cohort. **Table S5.** Percentage of missing data in the variables of interest in EDEN cohort. **Table S6.** BIC Fit statistics and model selection in the EDEN cohort. **Table S7.** Baseline characteristics between phenotypes in EDEN cohort. **Table S8.** Interaction between ARDS phenotypes and feeding management strategy for VFDs in EDEN cohort. **Table S9.** Accuracy of a Parsimonious probabilistic model in Correctly Identifying ARDS trajectories in EDEN cohort. **Figure S1.** The most important classifier variables on Day 0 from GBM in the FACTT cohort. **Figure S2.** Receiver operating curves (ROC) of the regression model for predicting phenotypes in the FACTT cohort.

## Data Availability

The datasets presented in the current study are available in the BioLINCC website. (https://biolincc.nhlbi.nih.gov).

## Abbreviations

ARDS: Acute respiratory distress syndrome
HTE: Heterogeneity of treatment effect
PFB: Positive fluid balance
NFB: Negative fluid balance
VFDs: Ventilator-free days
RCTs: Randomized controlled trials
MV: Mechanical ventilation
ICU: Intensive care unit
SOFA: Sequential Organ Failure Assessment
JLCMM: Joint latent class mixed model
BIC: Bayesian information criterion
AUROC: The area under the receiver operating characteristic curves
AUPRC: The area under the precision-recall curves

## References

[1] Meyer NJ, Gattinoni L, Calfee CS (2021). Acute respiratory distress syndrome. Lancet.

[2] McNicholas B, Madden MG, Laffey JG (2020). Machine learning classifier models: the future for acute respiratory distress syndrome phenotyping?. Am J Respir Crit Care Med.

[3] Sinha P, Delucchi KL, McAuley DF, O'Kane CM, Matthay MA, Calfee CS (2020). Development and validation of parsimonious algorithms to classify acute respiratory distress syndrome phenotypes: a secondary analysis of randomised controlled trials. Lancet Respir Med.

[4] Calfee CS, Delucchi K, Parsons PE, Thompson BT, Ware LB, Matthay MA (2014). Subphenotypes in acute respiratory distress syndrome: latent class analysis of data from two randomised controlled trials. Lancet Respir Med.

[5] Famous KR, Delucchi K, Ware LB, Kangelaris KN, Liu KD, Thompson BT (2017). Acute respiratory distress syndrome subphenotypes respond differently to randomized fluid management strategy. Am J Respir Crit Care Med.

[6] van Mourik N, Metske HA, Hofstra JJ, Binnekade JM, Geerts BF, Schultz MJ (2019). Cumulative fluid balance predicts mortality and increases time on mechanical ventilation in ARDS patients: an observational cohort study. PLoS ONE.

[7] Rosenberg AL, Dechert RE, Park PK, Bartlett RH (2009). Review of a large clinical series: association of cumulative fluid balance on outcome in acute lung injury: a retrospective review of the ARDSnet tidal volume study cohort. J Intensive Care Med.

[8] Woodward CW, Lambert J, Ortiz-Soriano V, Li Y, Ruiz-Conejo M, Bissell BD (2019). Fluid overload associates with major adverse kidney events in critically ill patients with acute kidney injury requiring continuous renal replacement therapy. Crit Care Med.

[9] Silversides JA, Major E, Ferguson AJ, Mann EE, McAuley DF, Marshall JC (2017). Conservative fluid management or deresuscitation for patients with sepsis or acute respiratory distress syndrome following the resuscitation phase of critical illness: a systematic review and meta-analysis. Intensive Care Med.

[10] Wiedemann HP, Wheeler AP, Bernard GR, Thompson BT, Hayden D, deBoisblanc B (2006). Comparison of two fluid-management strategies in acute lung injury. N Engl J Med.

[11] Rice TW, Wheeler AP, Thompson BT, Steingrub J, Hite RD, Moss M (2012). Initial trophic vs full enteral feeding in patients with acute lung injury: the EDEN randomized trial. JAMA.

[12] Proust-Lima C, Sene M, Taylor JM, Jacqmin-Gadda H (2014). Joint latent class models for longitudinal and time-to-event data: a review. Stat Methods Med Res.

[13] Sinha P, Churpek MM, Calfee CS (2020). Machine learning classifier models can identify acute respiratory distress syndrome phenotypes using readily available clinical data. Am J Respir Crit Care Med.

[14] Seymour CW, Kennedy JN, Wang S, Chang CH, Elliott CF, Xu Z (2019). Derivation, validation, and potential treatment implications of novel clinical phenotypes for sepsis. JAMA.

[15] Chen H, Yu Q, Xie J, Liu S, Pan C, Liu L (2022). Longitudinal phenotypes in patients with acute respiratory distress syndrome: a multi-database study. Crit Care.

[16] Mayr F, Tang L, Ou Y, Chang CH, Wang S, Kennedy JN (2020). Sepsis phenotypes are dynamic and associated with long-term outcomes. Am J Respir Crit Care Med.

[17] Wang MP, Jiang L, Zhu B, Du B, Li W, He Y (2021). Association of fluid balance trajectories with clinical outcomes in patients with septic shock: a prospective multicenter cohort study. Mil Med Res.

[18] Kuo G, Chen SW, Lee CC, Chen JJ, Fan PC, Wang SY (2020). Latent trajectories of fluid balance are associated with outcomes in cardiac and aortic surgery. Ann Thorac Surg.

[19] Schmidt M, Pham T, Arcadipane A, Agerstrand C, Ohshimo S, Pellegrino V (2019). Mechanical ventilation management during extracorporeal membrane oxygenation for acute respiratory distress syndrome. An international multicenter prospective cohort. Am J Respir Crit Care Med.

[20] Zinter MS, Spicer AC, Liu KD, Orwoll BE, Alkhouli MF, Brakeman PR (2019). Positive cumulative fluid balance is associated with mortality in pediatric acute respiratory distress syndrome in the setting of acute kidney injury. Pediatr Crit Care Med.

[21] Wiedemann HP, Wheeler AP, Bernard GR, Thompson BT, National Heart L, Blood Institute Acute Respiratory Distress Syndrome Clinical Trials N (2006). Comparison of two fluid-management strategies in acute lung injury. N Engl J Med.

[22] Bhavani SV, Carey KA, Gilbert ER, Afshar M, Verhoef PA, Churpek MM (2019). Identifying novel sepsis subphenotypes using temperature trajectories. Am J Respir Crit Care Med.

