# Supplementary material for: Identifying novel clinical phenotypes of acute respiratory distress syndrome using trajectories of daily fluid balance: a secondary analysis of randomized controlled trials
Source: Eur J Med Res. 2024 May 28;29:299. doi: 10.1186/s40001-024-01866-9 (PMC11134929; doi:10.1186/s40001-024-01866-9)

Identifying novel clinical phenotypes of acute respiratory distress syndrome using trajectories of fluid balance: a secondary analysis of randomized controlled trials

Additional file 1

**Method:**

**A**: Data Collection and outcomes;

**B**: Joint latent class mixed models (JLCMM);

**C**: Approach to missing Data

**Table S1**: Percentage of missing data in the variables of interest in FACTT cohort

Table S2: BIC Fit statistics and model selection in the FACTT cohort.

Table S3: Accuracy of a Parsimonious probabilistic model in Correctly Identifying ARDS trajectories in FACTT cohort.

Table S4: Interaction between ARDS phenotypes as defined by Probabilistic model and fluid management strategy for 60-day mortality in FACTT cohort.

Table S5: Percentage of missing data in the variables of interest in EDEN cohort

Table S6: BIC Fit statistics and model selection in the EDEN cohort.

Table S7: Baseline characteristics between phenotypes in EDEN cohort.

Table S8: Interaction between ARDS phenotypes and feeding management strategy for VFDs in EDEN cohort

Table S9: Accuracy of a Parsimonious probabilistic model in Correctly Identifying ARDS trajectories in EDEN cohort.

Figure S1: The most important classifier variables on Day 0 from GBM in the FACTT cohort.

Figure S2: Receiver operating curves (ROC) of the regression model for predicting phenotypes in the FACTT cohort.

**Method**

**A. Data Collection and outcomes**

In the development cohort, all demographic data, chronic comorbidities, hemodynamic and respiratory parameters, and laboratory values were collected at study baseline (pre-randomization). Severity of illness at baseline was assessed by Sequential Organ Failure Assessment (SOFA) score, Charlson Comorbidity index and acute lung injury score. We collected the fluid balance from Day 0 to Day 7 after randomization to develop the fluid balance trajectories, treatments and longitudinal clinical variables in the development cohort were also collected in the validation cohort, data were extracted as described for the development cohort. Day 0 was defined as the day prior to the randomization.

Longitudinal data of preselected variables were collected. These included hemodynamic variables (heart rate, systolic blood pressure, diastolic blood pressure, central venous pressure, use of vasopressor and pH), markers of organ dysfunction and injury (platelets, creatinine, bilirubin and PaO_2_/FiO_2_ ratio), and other variables (temperature, hemoglobin, sodium, glucose, and bicarbonate), all these variables were extracted from Day 0 until death, ICU discharge, or Day 7 in the ICU, whichever occurred first. Respiratory variables were also obtained, and included respiratory rate, tidal volume, minute ventilation, positive end–expiratory pressure (PEEP), peak pressure (Ppeak)， Plateau pressure (Pplat) and arterial PCO_2_, for patients who received ventilation in a volume–controlled assist mode, driving pressure (ΔP) was calculated as Pplat minus PEEP, if not specified, Ppeak was considered equal to Pplat in pressure-regulated modes other than pressure support ventilation. Mechanical power (MP) was calculated as [0·098*respiratory rate*tidal volume*(Ppeak–(0·5*ΔP))]. Ventilatory ratio is defined as [minute ventilation * arterial PCO_2_/ [predicted body weight (PBW) * 100 * 37.5], PBW was calculated as equal to [50 + 0.91 (centimeters of height – 152.4)] in males, and [45.5 + 0.91 (centimeters of height – 152.4)] in females. All the respiratory variables were obtained from Day 0 until death, ICU discharge, liberation from mechanical ventilation or Day 7 in the ICU, whichever occurred first.

Data of outcomes were also extracted. In the development cohort (FACTT)(1), The primary end point was 60-day mortality. In the validation cohort (EDEN) (2), the primary outcome was ventilator-free days (VFDs) through day 28, Mortality at day 60 after randomization was also collected

**B. Joint latent class mixed models (JLCMM)**

factt_jlcmm1<-Jointlcmm(fluid_balance~days*use_of_vasopressor,

random= ~days,

subject = "id",

survival = Surv(new_time,new_event)~age+gender+prior_bmi+primary_lung_injury

+base_pf, hazard = "Weibull", hazardtype = "PH",data=factt,ng=1)

factt_jlcmm2<-Jointlcmm(fluid_balance~days*use_of_vasopressor,

random= ~days,

subject = "id",

survival = Surv(new_time,new_event)~age+gender+prior_bmi+primary_lung_injury

+base_pf, hazard = "Weibull", hazardtype = "PH",data=factt,ng=2, B=factt_jlcmm)

factt_jlcmm3<-Jointlcmm(fluid_balance~days*use_of_vasopressor,

random= ~days,

subject = "id",

survival = Surv(new_time,new_event)~age+gender+prior_bmi+primary_lung_injury

+base_pf, hazard = "Weibull", hazardtype = "PH",data=factt,ng=3, B=factt_jlcmm)

factt_jlcmm4<-Jointlcmm(fluid_balance~days*use_of_vasopressor,

random= ~days,

subject = "id",

survival = Surv(new_time,new_event)~age+gender+prior_bmi+primary_lung_injury

+base_pf, hazard = "Weibull", hazardtype = "PH",data=factt,ng=4, B=factt_jlcmm)

factt_jlcmm5<-Jointlcmm(fluid_balance~days*use_of_vasopressor,

random= ~days,

subject = "id",

survival = Surv(new_time,new_event)~age+gender+prior_bmi+primary_lung_injury

+base_pf, hazard = "Weibull", hazardtype = "PH",data=factt,ng=5, B=factt_jlcmm)

summarytable(factt_jlcmm1,factt_jlcmm2,factt_jlcmm3,factt_jlcmm4,factt_jlcmm5)

**C.** **Approach to missing Data**

Missing data of baseline characteristics were summarized in Table S1 (Development cohort) and Table S8 (Validation cohort). We assumed that data were missing at random, and we used multiple imputation by chained equation (MICE) which generated values for all missing data using the observed data for all patients. In total we imputed 5 different data sets (n = 5), the imputation method was weighted predictive mean matching. We used ‘mice’ package in R studio to impute the data.

**References**

1. Rice TW, Wheeler AP, Thompson BT, Steingrub J, Hite RD, Moss M, Morris A, Dong N, Rock P. Initial trophic vs full enteral feeding in patients with acute lung injury: the EDEN randomized trial. Jama 2012; 307: 795-803.

2. Wiedemann HP, Wheeler AP, Bernard GR, Thompson BT, Hayden D, deBoisblanc B, Connors AF, Jr., Hite RD, Harabin AL. Comparison of two fluid-management strategies in acute lung injury. N Engl J Med 2006; 354: 2564-2575.

Table S1: Percentage of missing data in the variables of interest in FACTT cohort

| **FACTT cohort (N=992)** | |
| --- | --- |
| Age (years) | 0% |
| Male, n (%) | 0% |
| BMI (Kg/m^2^) | 8.1% |
| Ethnicity, n (%) | 0% |
| APACHE III score | 3.4% |
| Charlson Comorbidity index | 0% |
| ARDS Primary risk factor, n (%) | 0% |
| Respiratory rate (breaths min^-1^) | 0.8% |
| Tidal volume (ml) | 7.5% |
| FiO_2_ | 3.7% |
| Minute ventilation (L/min) | 3.5% |
| PEEP (cmH_2_0) | 0.9% |
| Peak Pressure (cmH_2_0) | 6.5% |
| Plateau pressure (cmH_2_0) | 13.2% |
| Driving pressure (cmH_2_0) | 13.5% |
| Mechanical power (J/min) | 15.5% |
| PaCO_2_ (mmHg) | 11.7% |
| PaO_2_/FiO_2_ ratio(mmHg) | 11.7% |
| Heart rate (beats min^-1^) | 0% |
| Systolic blood pressure (mmHg) | 0% |
| Diastolic blood pressure (mmHg) | 0.1% |
| MAP (mmHg) | 0.2% |
| Central venous pressure (mmHg) | 2.2% |
| Vasopressor use at baseline, n (%) | 0% |
| pH | 11.7% |
| Bicarbonate (mmol/L) | 1.2% |
| Blood urea nitrogen (mg/dl) | 1.4% |
| Creatinine (mg/dl) | 1.1% |
| Platelets (*10^9^/L) | 0.4% |
| White cell count (*10^9^/L) | 1.2% |
| Albumin (g/dl) | 13.0% |
| Bilirubin (mg/dl) | 26.4% |
| Temperature (°C) | 0.2% |
| Sodium (mmol/L) | 0.5% |
| Glucose (mg/dl) | 0.9% |
| Fluid balance (L over previous 24 h) | 0% |

Table S2: BIC Fit statistics and model selection in the FACTT cohort.

| Trajectories | BIC | Group membership | | | | |
| --- | --- | --- | --- | --- | --- | --- |
| 2 | 22099.96 | 26.4% | 73.6% | — | — | — |
| 3 | 22052.44 | 73.4% | 2.0% | 24.6% | — | — |
| 4 | 22074.90 | 0.8% | 1.9% | 72.4% | 24.9% | — |
| 5 | 2073.60 | 1.01% | 13.7% | 17.4% | 1.3% | 66.5% |

BIC: Bayesian information criterion

Table S3: Accuracy of a Parsimonious probabilistic model in Correctly Identifying ARDS trajectories in FACTT cohort.

|  | **Class 1**  **(Joint LCMM)** | **Class 2**  **(Joint LCMM)** |
| --- | --- | --- |
| **Class 1**  **(****Probabilistic model)** | 605  (79.8%) | 87  (37.2%) |
| **Class 2**  **(Probabilistic model)** | 153  (20.2%) | 147  (62.8%) |
|  | 758 | 234 |

Table S4: Interaction between ARDS phenotypes as defined by Probabilistic model and fluid management strategy for 60-day mortality in FACTT cohort.

| Fluid-management strategy | Class 1 (n=706) | | Class 2 (n=286) | | P value for interaction |
| --- | --- | --- | --- | --- | --- |
|  | Conservative  (n=430) | Liberal  (n=314) | Conservative  (n=68) | Liberal  (n=180) |  |
| 60-day mortality, n (%) | 17.7 | 16.2 | 69.1 | 48.3 | 0.07 |

Table S5: Percentage of missing data in the variables of interest in EDEN cohort

| **EDEN cohort (N=998)** | |
| --- | --- |
| Age (years) | 0% |
| Male | 0% |
| BMI (Kg/m^2^) | 0.3% |
| Ethnicity | 0% |
| Charlson Comorbidity index | 0% |
| ARDS Primary risk factor | 0% |
| Respiratory rate (breaths min^-1^) | 0.6% |
| Tidal volume (ml) | 12.3% |
| FiO_2_ | 3.4% |
| PEEP (cmH_2_0) | 1.0% |
| Peak Pressure (cmH_2_0) | 22.6% |
| Plateau pressure (cmH_2_0) | 22.6% |
| Driving pressure (cmH_2_0) | 23.5% |
| Mechanical power (J/min) | 28.9% |
| PaCO_2_ (mmHg) | 2.8% |
| PaO_2_/FiO_2_ (mmHg) | 3.4% |
| Heart rate (beats min^-1^) | 0% |
| Systolic blood pressure (mmHg) | 0.1% |
| Diastolic blood pressure (mmHg) | 0% |
| MAP (mmHg) | 12.1% |
| Vasopressor use at baseline | 0% |
| pH | 2.8% |
| Bicarbonate | 0.5% |
| Blood urea nitrogen (mg/dl) | 0.4% |
| Creatinine (mg/dl) | 0.2% |
| Urine output (L over previous 24 h) | 0.4% |
| Hemoglobin (g/dl) | 0.5% |
| Platelets (*10^9^/L) | 0.5% |
| White cell count (*10^9^/L) | 0.3% |
| Albumin (g/dl) | 3.6% |
| Bilirubin (mg/dl) | 8.0% |
| Temperature (°C) | 0.1% |
| Sodium (mmol/L) | 0.4% |
| Glucose (mg/dl) | 0.3% |
| Fluid balance (L over previous 24 h) | 0.5% |

Table S6: BIC Fit statistics and model selection in the EDEN cohort.

| Trajectories | BIC | Group membership | | | | |
| --- | --- | --- | --- | --- | --- | --- |
| 2 | 22468.74 | 18.6% | 81.4% | — | — | — |
| 3 | 22470.71 | 29.7% | 3.3% | 67.0% | — | — |
| 4 | 22483.35 | 11.5% | 22.9% | 3.9% | 61.6% | — |
| 5 | 22481.01 | 1.1% | 54.8% | 13.8% | 4.0% | 26.2% |

BIC: Bayesian information criterion

Table S7: Baseline characteristics between phenotypes in EDEN cohort.

|  | All  (n=998) |  | | |
| --- | --- | --- | --- | --- |
|  |  | Class 1  (n=790) | Class 2  (n=208) | P value |
| Age (years) | 52 (42, 63) | 52 (41, 62) | 52 (45, 65) | 0.105 |
| Male (gender), n (%) | 509 (51) | 397 (50.3) | 112 (53.8) | 0.398 |
| BMI (kg/m^2^) | 28.8 (24.0, 34.7) | 29.3 (24.2, 34.9) | 27.6 (23.1, 33.9) | 0.042 |
| ARDS Primary risk factor, n (%) | | | | 0.949 |
| Pneumonia | 649 (65) | 513 (64.9) | 136 (65.4) |  |
| Sepsis | 146 (14.6) | 117 (14.8) | 29 (13.9) |  |
| Aspiration | 96 (9.6) | 79 (10) | 17 (8.2) |  |
| Other | 107 (10.7) | 81 (10.3) | 26 (12.5) |  |
| Charlson comorbidity index | 1 (0, 2) | 1 (0, 2) | 1 (0, 2) | 0.318 |
| Severity of ARDS at baseline, n (%) | | | | 0.010 |
| Mild | 223 (23.1) | 183 (24) | 40 (19.8) |  |
| Moderate | 494 (51.2) | 395 (51.8) | 99 (49) |  |
| Severe | 247 (25.6) | 184 (24.1) | 63 (31.2) |  |
| Parameters of mechanical ventilation in the first 24 h | | | | |
| Respiratory rate (breaths min^-1^) | 25 (20, 30) | 25 (20, 30) | 26 (22, 30.75) | 0.039 |
| Tidal volume (ml/kg PBW) | 6.1 (6.0, 6.8) | 6.1 (6.0, 6.7) | 6.1 (6.0, 6.9) | 0.251 |
| PEEP (cmH_2_0) | 10 (5, 12) | 10 (5, 12) | 10 (6, 12) | 0.237 |
| Plate Pressure (cmH_2_0) | 23 (19, 27) | 23 (19, 27) | 22 (19, 27) | 0.321 |
| Driving pressure (cmH_2_0) | 13 (10, 17) | 13 (10, 17) | 13 (10, 16) | 0.3 |
| Mechanical power (J/min) | 20.4 (14.5, 27.9) | 20.1 (14.4, 27.8) | 21.5 (15.2, 28.2) | 0.238 |
| PaCO_2_ (mmHg) | 38 (34, 45) | 38 (34, 45) | 38 (33, 46) | 0.609 |
| PaO_2_/FiO_2_ ratio (mmHg) | 139 (100, 196) | 140 (103, 198) | 130 (92, 181) | 0.017 |
| Vasopressor use in the first 24h, n (%) | 381 (38.2) | 274 (34.6) | 107 (51.4) | <0.001 |
| Vital signs in the first 24 h | | | | |
| Heart rate (beats min^-1^) | 94 (81, 108) | 93 (80, 107) | 98 (85, 110) | 0.018 |
| MAP (mmHg) | 74 (67, 82) | 76 (68, 83) | 72 (66, 80) | 0.036 |
| Temperature (℃) | 37.3 (36.8, 37.9) | 37.3 (36.8, 37.9) | 37.2 (36.7, 38) | 0.424 |
| Laboratory data in the first 24 h | | | | |
| pH | 7.36 (7.3, 7.42) | 7.36 (7.31, 7.42) | 7.34 (7.29, 7.39) | 0.034 |
| BUN (mg/dl) | 21 (13, 36) | 20 (12, 35) | 23 (16, 38.25) | 0.005 |
| Creatinine (mg/dL) | 1 (0.7, 1.6) | 1 (0.7, 1.5) | 1.1 (0.8, 1.7) | 0.039 |
| Bicarbonate (mmol/L) | 22 (19, 26) | 23 (20, 26) | 22 (19, 24) | 0.005 |
| Platelet (*10^9^/L) | 170 (109, 242) | 175 (110, 243) | 152 (103, 222) | 0.048 |
| Fluid balance in the first 24h (L) | 2.02 (4.3, 3.95) | 1.92 (0.32, 3.88) | 2.28 (0.80-4.13) | 0.023 |
| Alive and VFDs at Day 28 (days) | 19 (0, 23) | 21 (0.3, 24) | 0 (0, 18) | < 0.001 |
| 60-day mortality, n (%) | 225 (22.5) | 149 (18.9) | 76 (36.5) | <0.001 |

ICU: intensive care unit; ARDS: acute respiratory distress syndrome; FiO_2_: fraction of inspired oxygen; PEEP: positive end-expiratory pressure; PaCO_2_: partial pressure of Carbon Dioxide; PaO_2_: partial pressure of oxygen; MAP: mean arterial blood pressure.

Table S8: Interaction between ARDS phenotypes and feeding management strategy for VFDs in EDEN cohort

| Fluid-management strategy | Class 1 (n=790) | | Class 2 (n=208) | | P value for interaction |
| --- | --- | --- | --- | --- | --- |
|  | Full  (n=368) | Trophic  (n=422) | Full  (n=123) | Trophic  (n=85) |  |
| Ventilator-free days, days | 20 (9, 24) | 21 (0, 24) | 1 (0, 18) | 0 (0, 17) | 0.902 |

Table S9: Accuracy of a Parsimonious probabilistic model in Correctly Identifying ARDS trajectories in EDEN cohort.

|  | **Class 1**  **(Joint LCMM)** | **Class 2**  **(Joint LCMM)** |
| --- | --- | --- |
| **Class 1**  **(Probabilistic model)** | 542 (68.6%) | 98 (47.1%) |
| **Class 2**  **(Probabilistic model)** | 248 (31.4%) | 110 (52.9%) |
|  | 790 | 208 |

Figure S1: **The most important classifier variables on Day 0 from GBM in the FACTT cohort.**


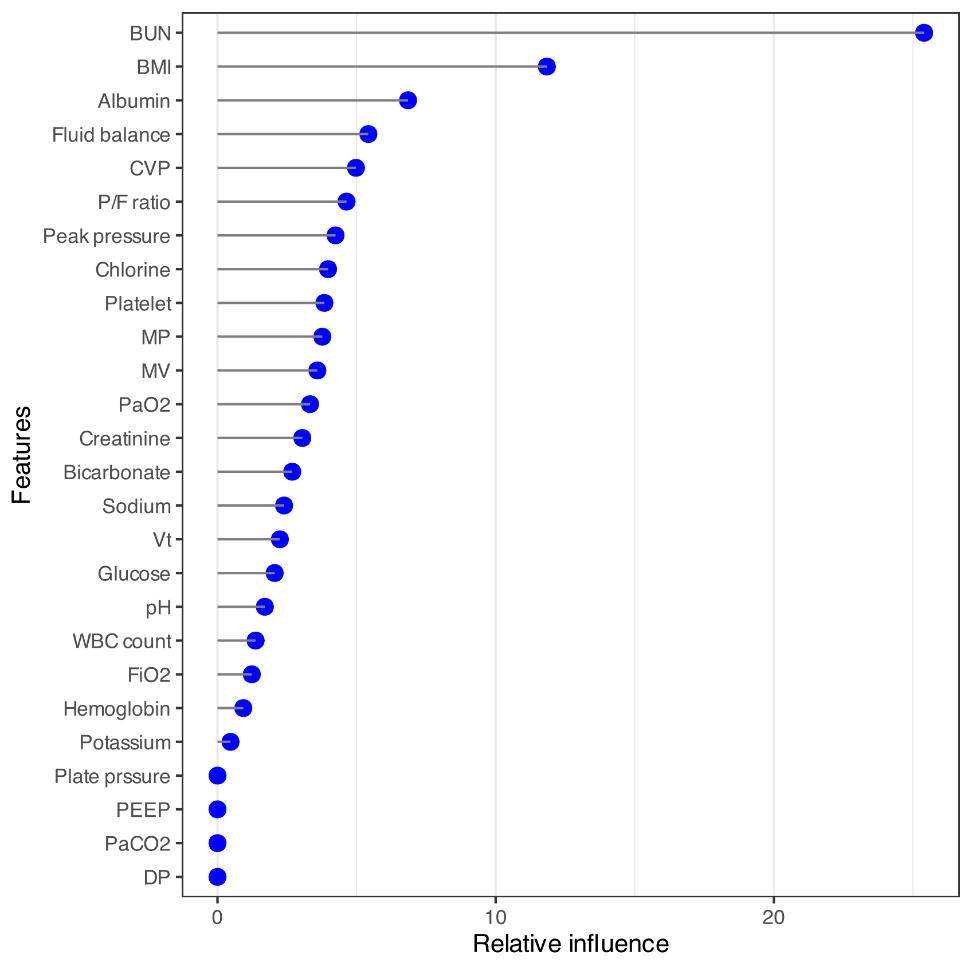


BUN: Blood urea nitrogen; BMI: body mass index; CVP: Central venous pressure; P/F ratio: partial pressure of oxygen/ fraction of inspired oxygen ratio; MP: mechanical power; MV: minute volume; PaO_2_: partial pressure of oxygen; Vt: Tidal volume; FiO_2_: fraction of inspired oxygen; PEEP: positive end-expiratory pressure; PaCO_2_: partial pressure of Carbon Dioxide; DP: Driving pressure.

Figure S2: Receiver operating curves (ROC) of the regression model for predicting phenotypes in the FACTT cohort.


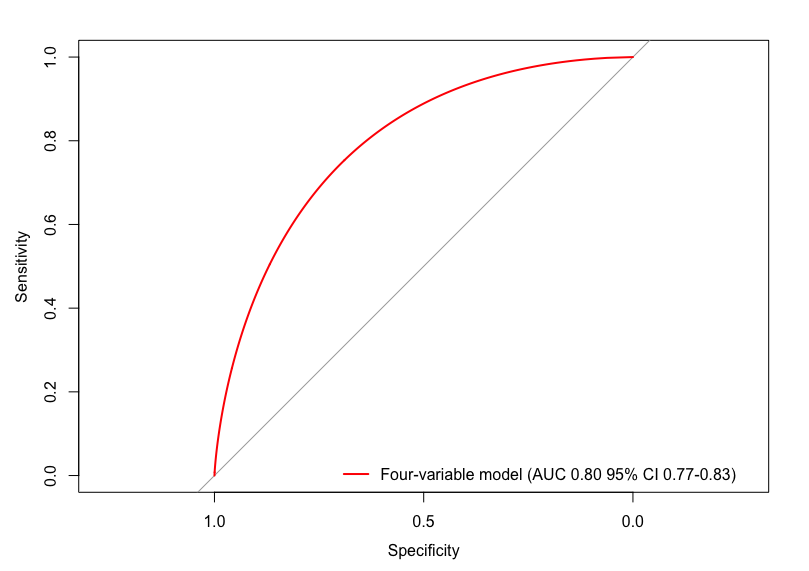

Supplement: Supplementary file 1 — Additional file 1: Table S1. Percentage of missing data in the variables of interest in FACTT cohort. Table S2. BIC Fit statistics and model selection in the FACTT cohort. Table S3. Accuracy of a Parsimonious probabilistic model in Correctly Identifying ARDS trajectories in FACTT cohort. Table S4. Interaction between ARDS phenotypes as defined by Probabilistic model and fluid management strategy for 60-day mortality in FACTT cohort. Table S5. Percentage of missing data in the variables of interest in EDEN cohort. Table S6. BIC Fit statistics and model selection in the EDEN cohort. Table S7. Baseline characteristics between phenotypes in EDEN cohort. Table S8. Interaction between ARDS phenotypes and feeding management strategy for VFDs in EDEN cohort. Table S9. Accuracy of a Parsimonious probabilistic model in Correctly Identifying ARDS trajectories in EDEN cohort. Figure S1. The most important classifier variables on Day 0 from GBM in the FACTT cohort. Figure S2. Receiver operating curves (ROC) of the regression model for predicting phenotypes in the FACTT cohort. [file 40001_2024_1866_MOESM1_ESM.docx]
